# Supplementary material for: DECODE: a Deep-learning framework for Condensing enhancers and refining boundaries with large-scale functional assays
Source: Bioinformatics. 2021 Jul 12;37(Suppl 1):i280–8. doi: 10.1093/bioinformatics/btab283 (PMC8275369; doi:10.1093/bioinformatics/btab283)
Supplement: btab283_Supplementary_Data [file btab283_supplementary_data.zip › btab283-suppl_data/gerstein.40.supp.pdf]

Supplementary text and figures for

“DECODE: A Deep-learning Framework for Condensing Enhancers and Refining Boundaries with Large-scale Functional Assays”

Table of Contents

1 PhastCons Scores 2

2 Rare DAF Enrichment 2

3 NPC Prediction LDSC 3

4 Model Comparison 3

5 QTL Enrichment 4

6 Chromatin Accessibility Displacement and Removal 5

1 PhastCons Scores

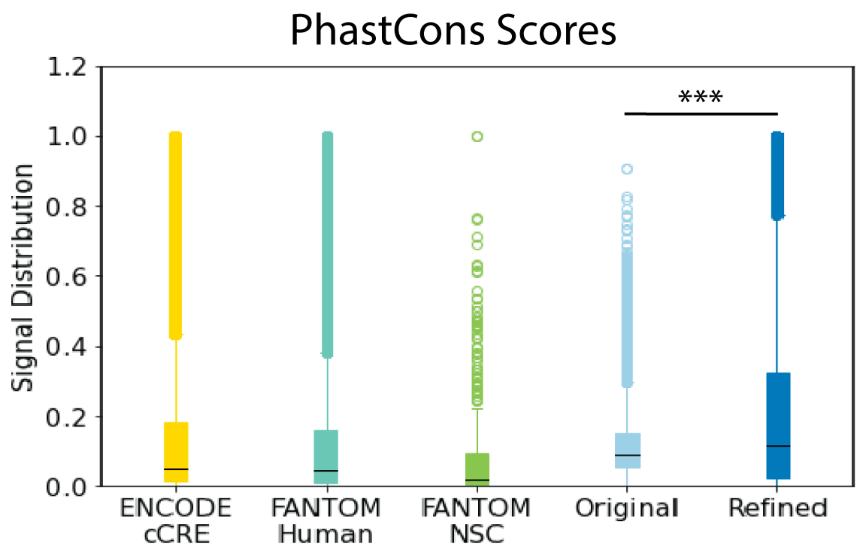

Supplemental Figure 1: 100-way PhastCons score distribution of original and refined set compared to ENCODE cCREs, FANTOM Human Enhancers and FANTOM Neuronal Stem Cell Differentially Expressed Enhancers.

2 Rare DAF Enrichment

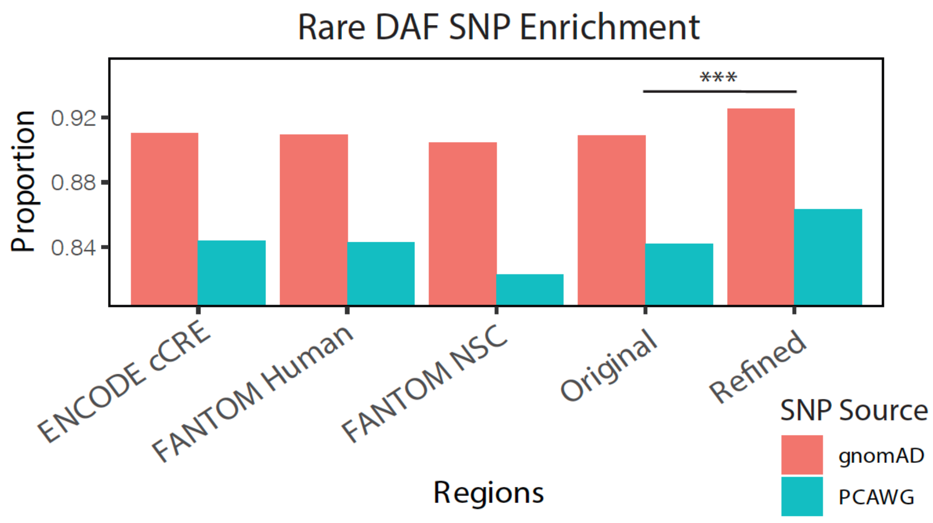

Supplemental Figure 2: Rare DAF SNP Enrichment of original and refined set compared to ENCODE cCREs, FANTOM Human Enhancers and FANTOM Neuronal Stem Cell Differentially Expressed Enhancers.

3 NPC Prediction LDSC

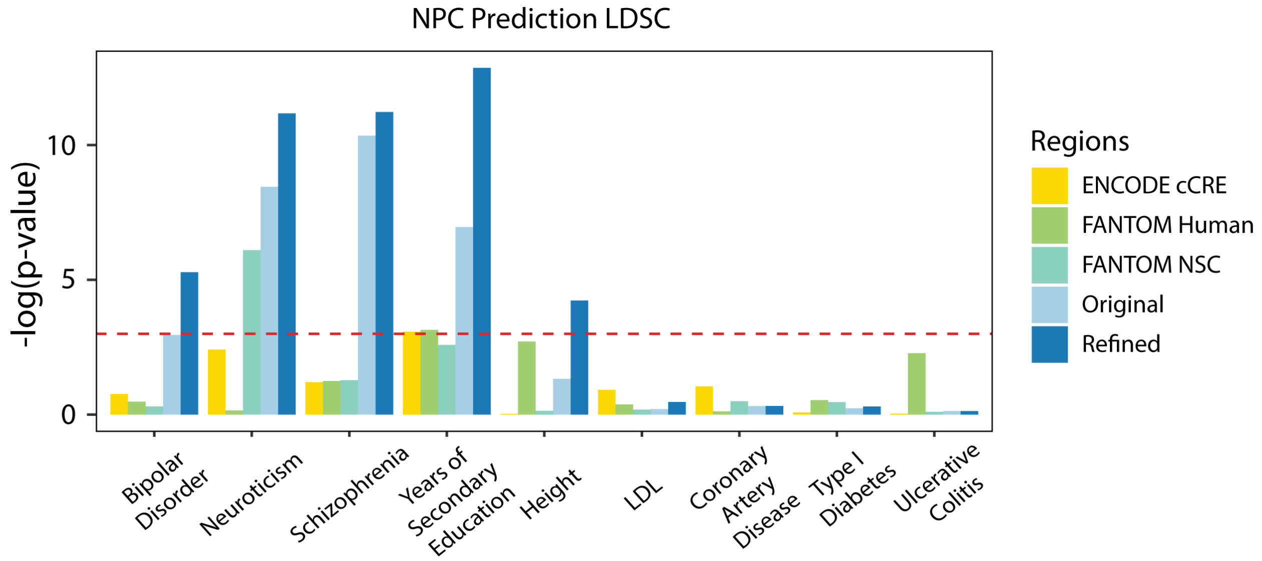

Supplemental Figure 3: Stratified LDSC Enrichment of original and refined set compared to ENCODE cCREs, FANTOM Human Enhancers and FANTOM Neuronal Stem Cell Differentially Expressed Enhancers.

4 Model Comparison

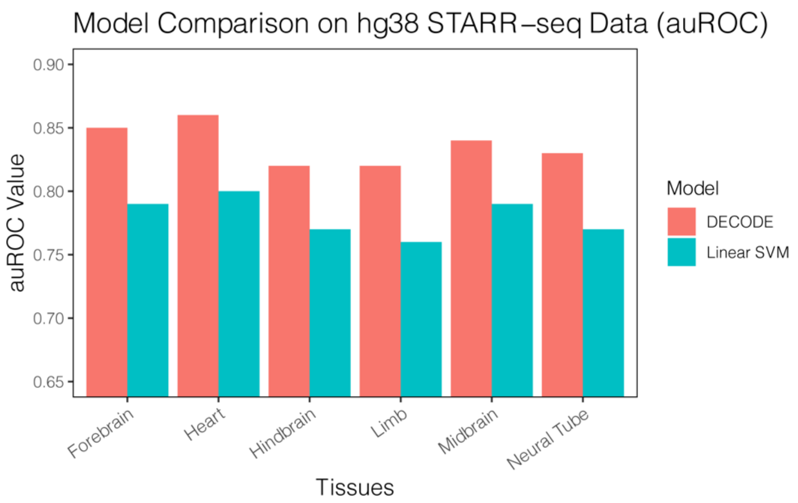

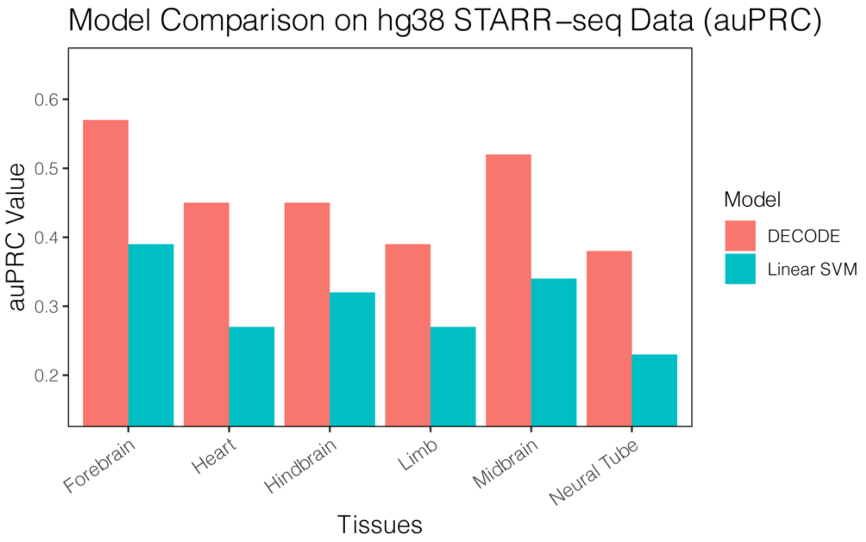

Supplemental Figure 4 and 5: Validation auROC and auPRC of our DECODE model compared to a linear SVM that resembles the Matched-Filter model. Both models were trained on the same human STARR-seq dataset and evaluated on validated mouse enhancers.

5 QTL Enrichment

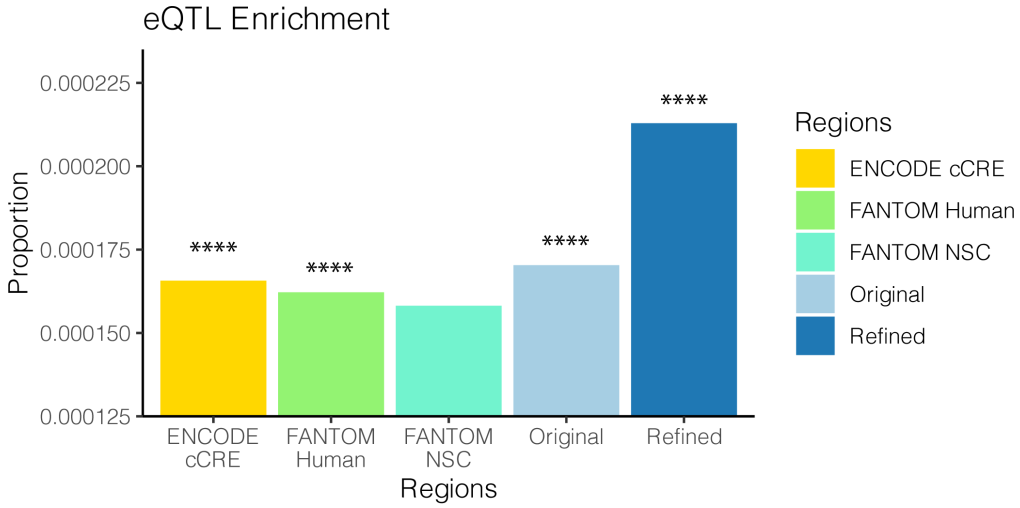

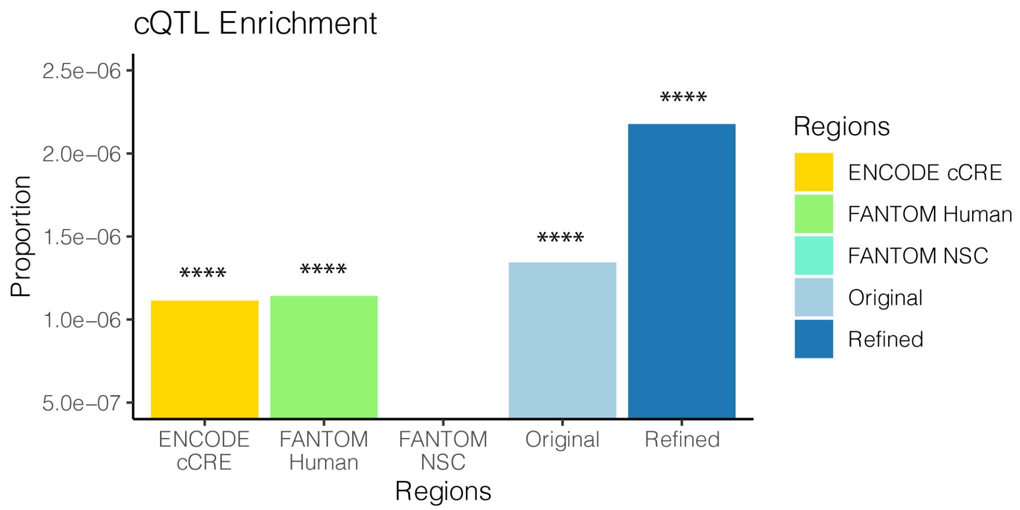

Supplemental Figure 6 and 7: auROC and auPRC of our DECODE model compared to a linear SVM that resembles the Matched-Filter model. Both models were trained on the same human STARR-seq dataset and evaluated on validated mouse enhancers.

6 LDSC Effect Size

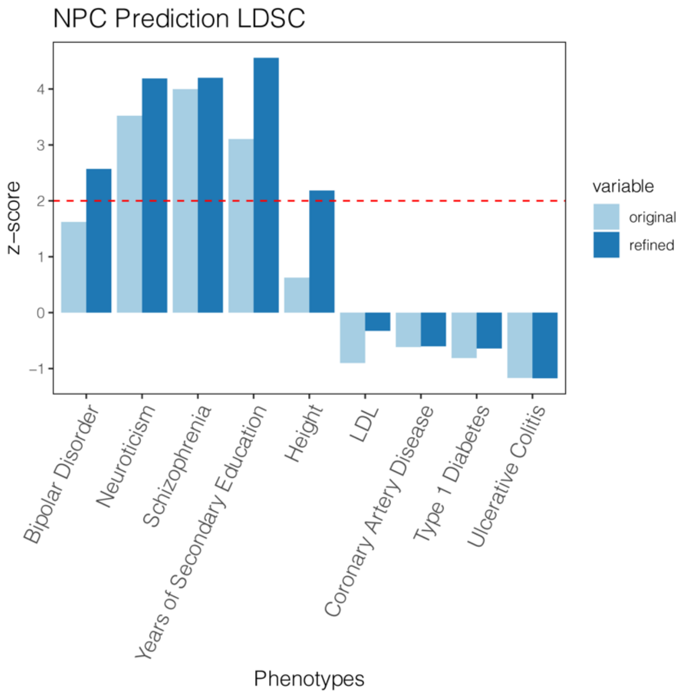

Supplemental Figure 8: The effect sizes of stratified LDSC enrichment for Figure 7(d).

7 Chromatin Accessibility Displacement and Removal

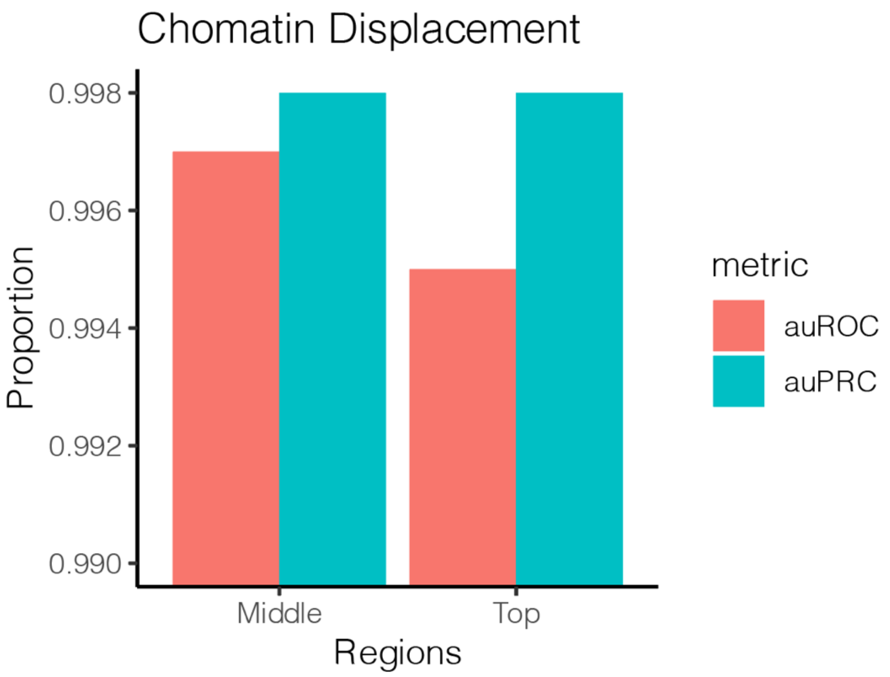

Supplemental Figure 9: We permutated the relative positive of chromatin accessibility signals to evaluate its effect on prediction outcomes. Chromatin accessibility signals were moved from the middle to the top of the input matrix. When evaluated on out-of-sample mouse enhancers, we see no significant decrease in validation metrics compared to our control model.

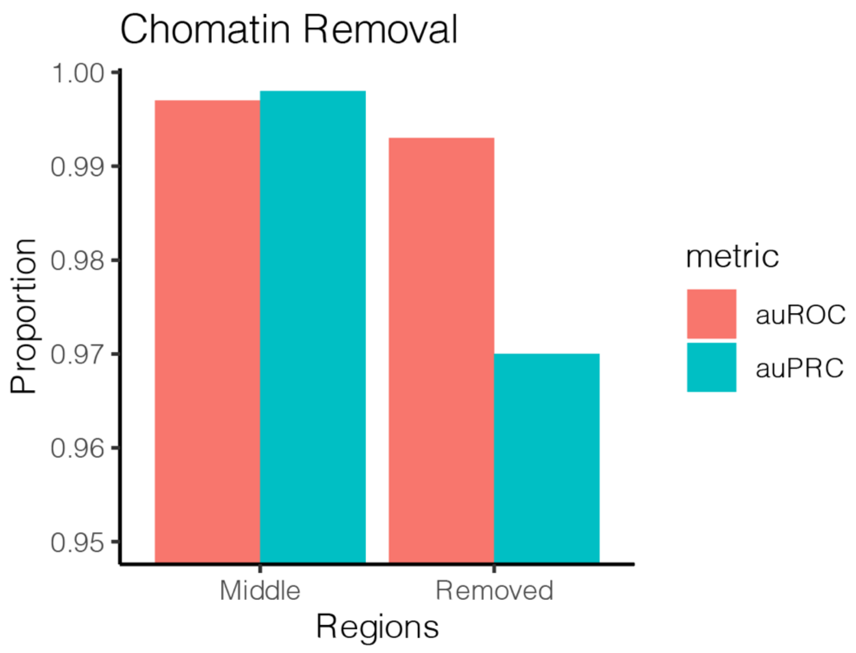

Supplemental Figure 10: We removed chromatin accessibility from out input matrix to evaluate its effect on prediction outcomes. Chromatin accessibility signals were removed from the input matrix, and the CNN architecture was also altered to adjust for the change in input dimension. When evaluated on out-of-sample mouse enhancers, we see a 2-5% decrease in validation metrics compared to our control model with chromatin accessibility.
