# Supplementary figures and images for: DECODE: a Deep-learning framework for Condensing enhancers and refining boundaries with large-scale functional assays
Source: Bioinformatics. 2021 Jul 12;37(Suppl 1):i280–8. doi: 10.1093/bioinformatics/btab283 (PMC8275369; doi:10.1093/bioinformatics/btab283)

# PhastCons Scores

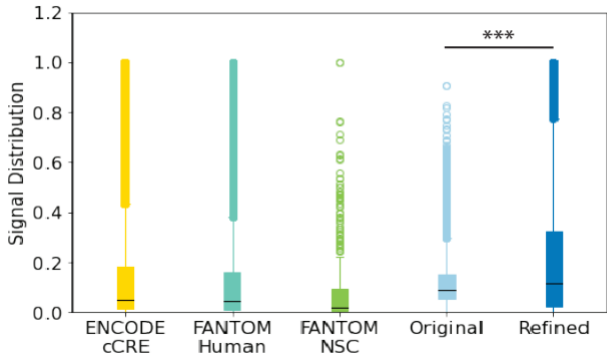

Supplement: btab283_Supplementary_Data [file btab283_supplementary_data.zip › btab283-suppl_data/gerstein.40.supp.1.pdf]

# Chomatin Removal

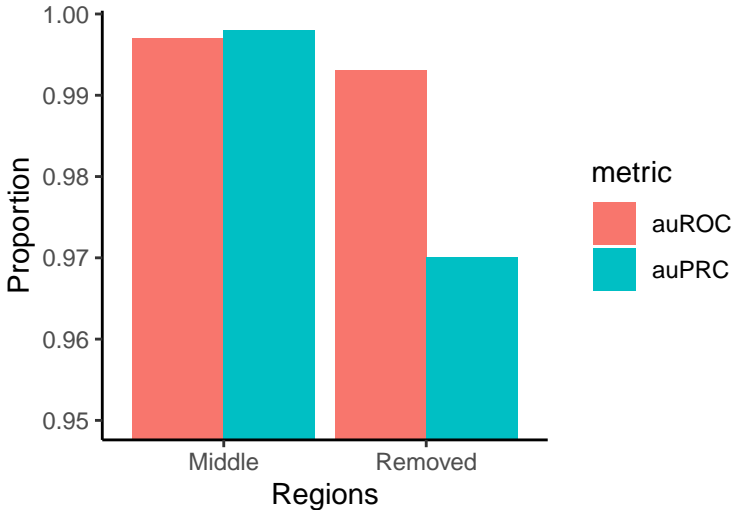

Supplement: btab283_Supplementary_Data [file btab283_supplementary_data.zip › btab283-suppl_data/gerstein.40.supp.10.pdf]

## Rare DAF SNP Enrichment

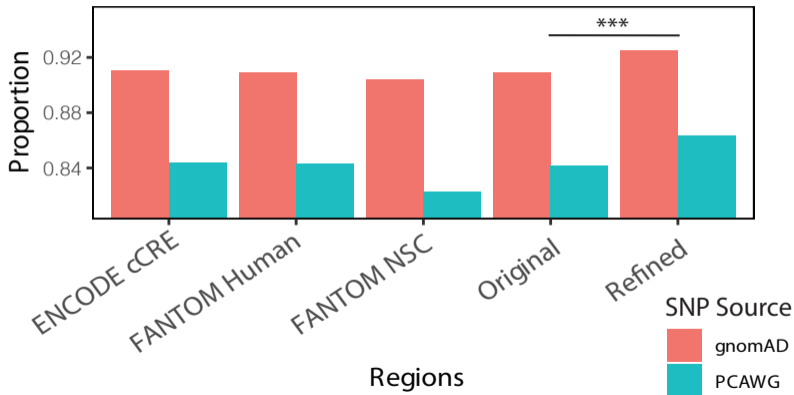

Supplement: btab283_Supplementary_Data [file btab283_supplementary_data.zip › btab283-suppl_data/gerstein.40.supp.2.pdf]

## NPC Prediction LDSC

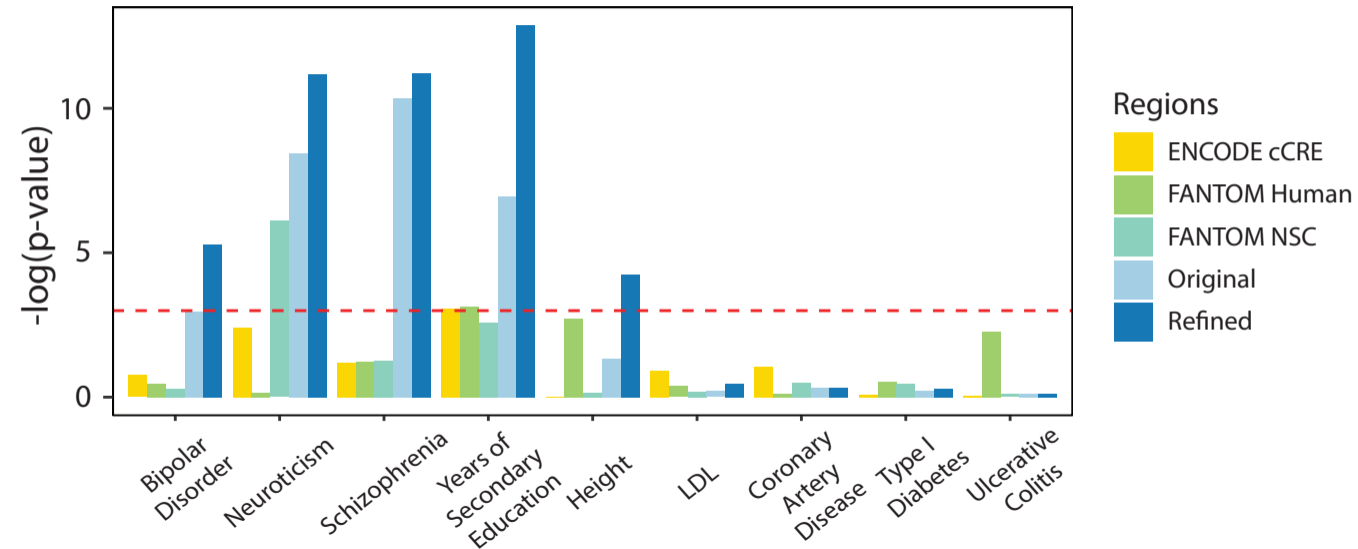

Supplement: btab283_Supplementary_Data [file btab283_supplementary_data.zip › btab283-suppl_data/gerstein.40.supp.3.pdf]

# Model Comparison on hg38 STARR-seq Data (auPRC)

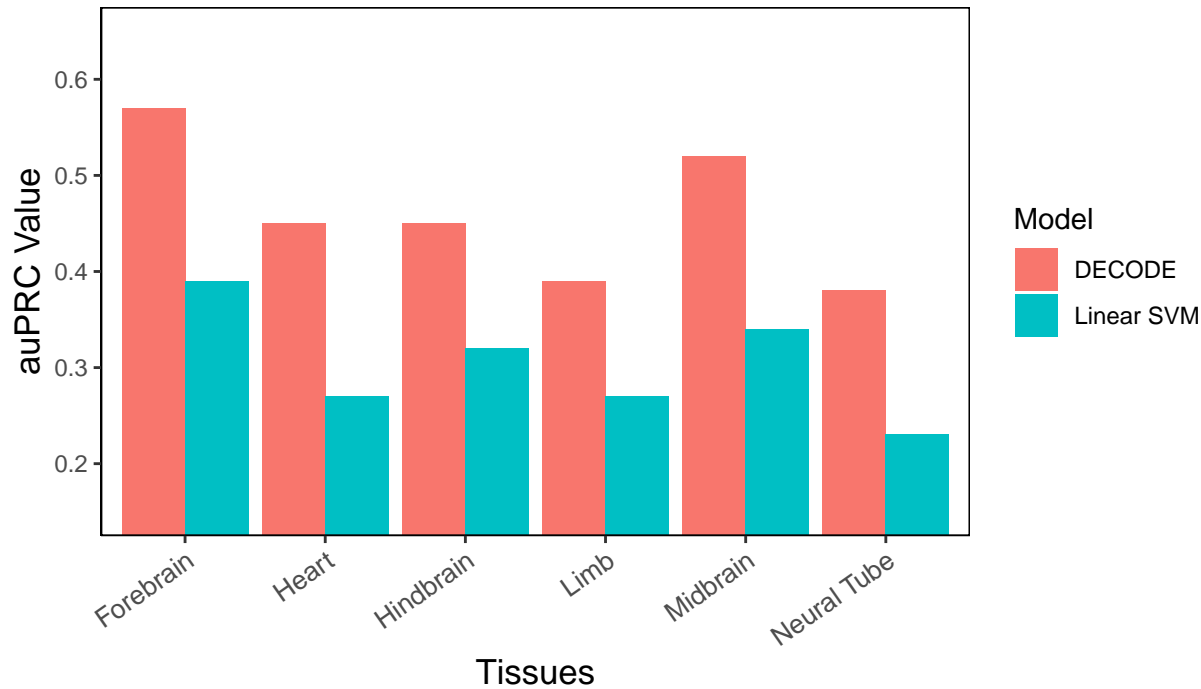

Supplement: btab283_Supplementary_Data [file btab283_supplementary_data.zip › btab283-suppl_data/gerstein.40.supp.4.pdf]

# Model Comparison on hg38 STARR-seq Data (auROC)

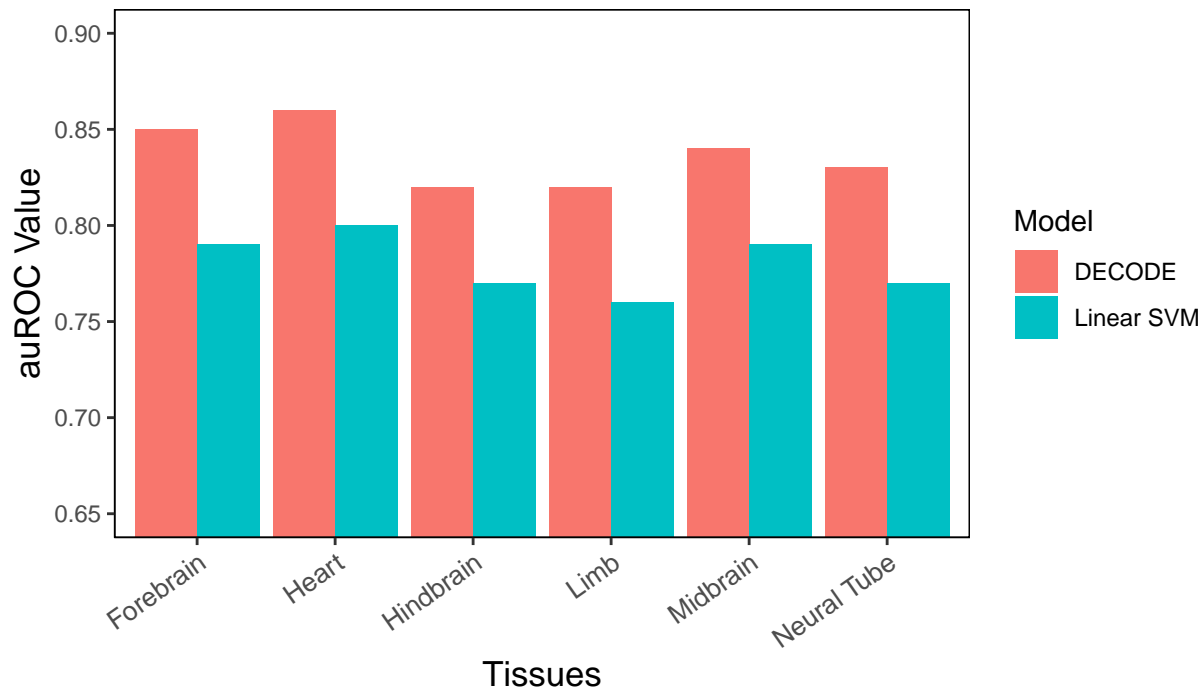

Supplement: btab283_Supplementary_Data [file btab283_supplementary_data.zip › btab283-suppl_data/gerstein.40.supp.5.pdf]

# eQTL Enrichment

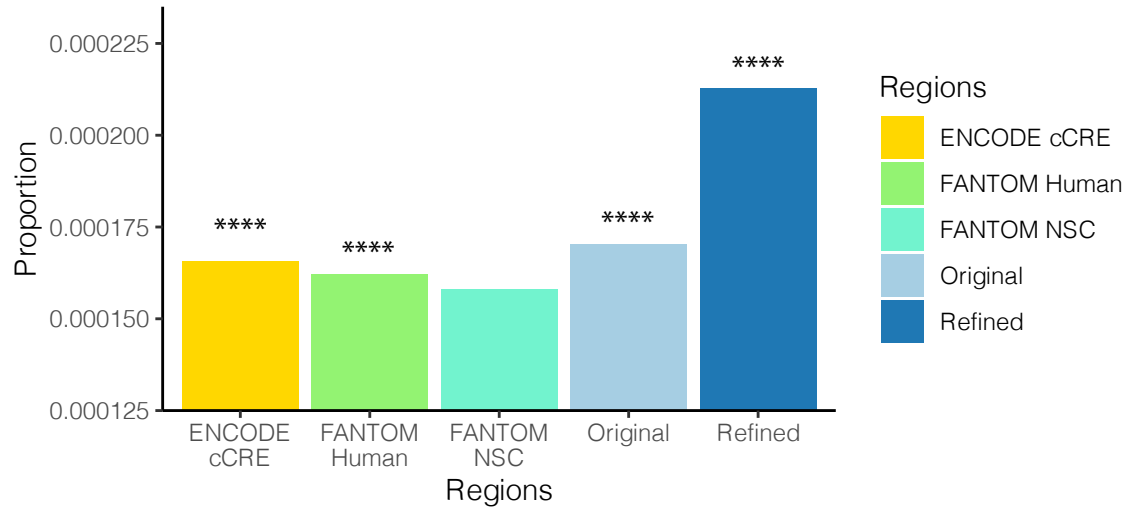

Supplement: btab283_Supplementary_Data [file btab283_supplementary_data.zip › btab283-suppl_data/gerstein.40.supp.6.pdf]

# cQTL Enrichment

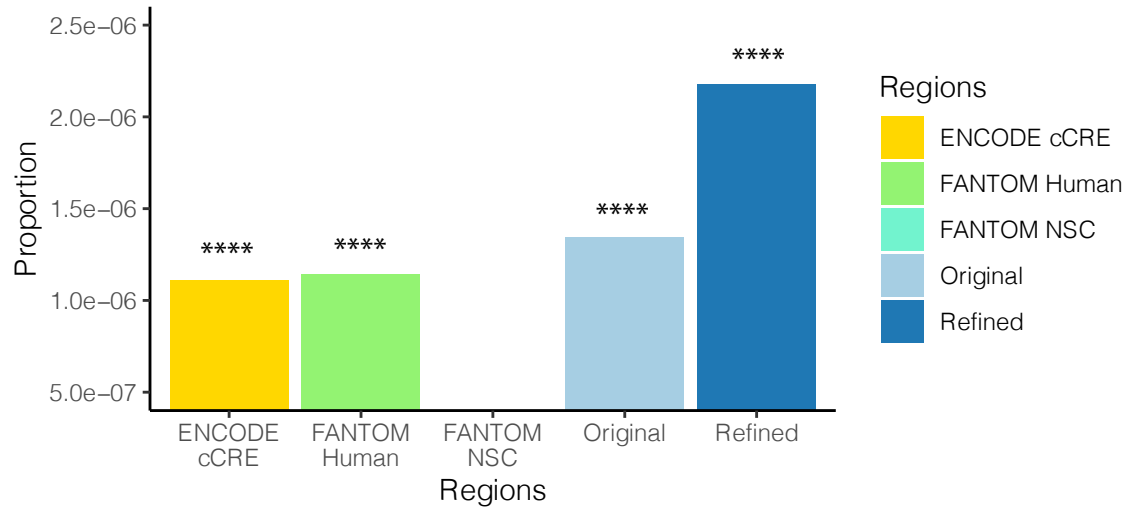

Supplement: btab283_Supplementary_Data [file btab283_supplementary_data.zip › btab283-suppl_data/gerstein.40.supp.7.pdf]

# NPC Prediction LDSC

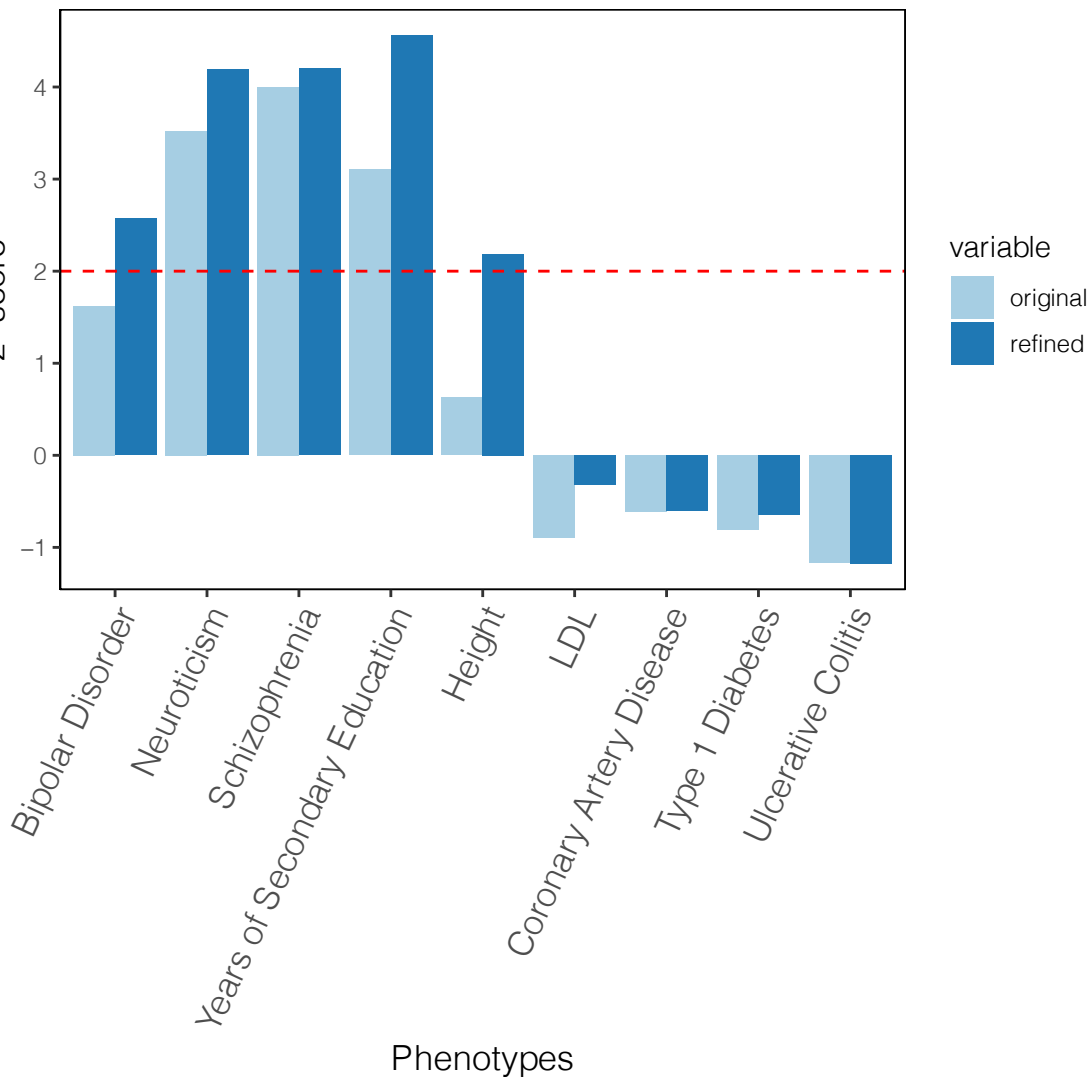

Supplement: btab283_Supplementary_Data [file btab283_supplementary_data.zip › btab283-suppl_data/gerstein.40.supp.8.pdf]

# Chomatin Displacement

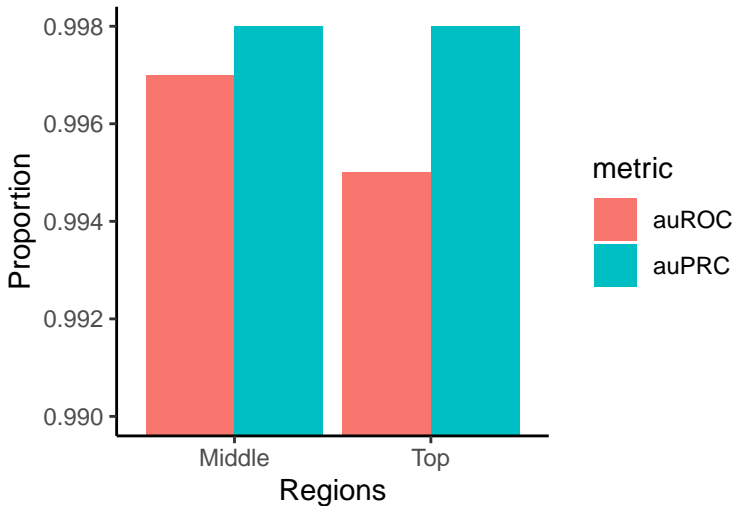

Supplement: btab283_Supplementary_Data [file btab283_supplementary_data.zip › btab283-suppl_data/gerstein.40.supp.9.pdf]
